# When the robot bleeds: risk factors and outcomes of intraoperative blood loss in robotic liver resection

**DOI:** 10.1007/s13304-026-02596-9

**Published:** 2026-03-24

**Authors:** Silvio Caringi, Antonella Delvecchio, Rebecca Marino, Paolo Magistri, Andrea Belli, Annarita Libia, Graziano Ceccarelli, Francesco Izzo, Marcello Giuseppe Spampinato, Nicola de’ Angelis, Patrick Pessaux, Tullio Piardi, Fabrizio Di Benedetto, Francesca Ratti, Riccardo Memeo

**Affiliations:** 1 Department of Surgery, Università Degli Studi Roma “Tor Vergata”, Via Montpellier, 1, 00133 Rome, Italy; 2 Unit of Hepato-Biliary and Pancreatic Surgery, “F. Miulli” General Hospital, Acquaviva delle Fonti, 70021 Bari, Italy; 3 Department of Medicine and Surgery, LUM University, 70010 Casamassima, Bari, Italy; 4 Hepatobiliary Surgery Division, IRCCS San Raffaele Scientific Institute, 20132 Milano, Italy; 5 Unit of Hepato-Pancreato-Biliary Surgery and Liver Transplantation, University of Modena and Reggio Emilia, 41121 Modena, Italy; 6 Unit of Hepato-Biliary and Pancreatic Surgery, Istituto Nazionale Tumori IRCCS Fondazione G. Pascale, 80131 Napoli, Italy; 7 Unit of General Surgery, “Vito Fazzi” Hospital, 73100 Lecce, Italy; 8 Unit of General Surgery, San Giovanni Battista Hospital, USL Umbria 2, 06034 Foligno, Italy; 9 Department of Surgery, Fondazione Poliambulanza, Brescia, Italy; 10 Department of Translational Medicine and LTTA Centre, University of Ferrara, Via Aldo Moro 8, 44124 Ferrara, Italy; 11 Department of Visceral and Digestive Surgery, Unit of Hepato-Bilio-Pancreatic Surgery, Nouvel Hospital Civil, University Hospital of Strasbourg, Inserm U1110, 67000 Strasbourg, France; 12 Unit of Surgery, Hôpital Robert Debré, 51100 Reims, France; 13 Hepatobiliary Surgery Division, Vita-Salute San Raffaele University, 20132 Milano, Italy

**Keywords:** Robotic liver resection, Intraoperative blood loss, Cirrhosis, Surgical complexity, Multicenter study

## Abstract

**Background:**

Despite the advantages of robotic-assisted liver resection (RALR), intraoperative blood loss (IBL) remains a main concern in hepatic surgery. The current multicenter study was designed to evaluate the determinants of significant intraoperative bleeding in RALR and the clinical impact of such bleeding.

**Methods:**

This study retrospectively analyzed 708 consecutive RALR performed in nine high-volume European centers between 2011 and 2023. All demographic, intraoperative, and postoperative data were extracted from a shared database. Patients were stratified into two groups based on the IBL value (< 500 mL vs. ≥ 500 mL). Variables that reached the level of *p* < 0.10 at univariable analysis were included in a multivariate logistic regression model.

**Results:**

The mean IBL was 224.7 mL, and 9.6% of patients experienced IBL ≥ 500 mL. Both cirrhosis (OR 3.00, 95% CI 1.63–5.52; *p* < 0.001) and higher TAMPA score (OR 1.09 per point, 95% CI 1.03–1.16; *p* = 0.004) independently predicted major bleeding. Patients with IBL ≥ 500 mL had longer operative times (372 vs. 239 min, *p* < 0.001), higher morbidity (72% vs. 46%, *p* < 0.001), and greater mortality (4.4% vs. 0.6%, *p* = 0.022).

**Conclusions:**

During RALR significant bleeding persists in 10% of cases. Cirrhosis and procedural complexity remain independent predictors. Early identification of the high-risk patient and tailored perioperative strategies are of paramount importance in reducing bleeding and improving overall outcomes following robotic hepatobiliary surgery.

## Introduction

Minimally invasive liver surgery has become the standard of care for selected patients undergoing hepatic resection, ensuring reduced postoperative morbidity and comparable oncological outcomes to open surgery [1–3]. Robot-assisted liver resection (RALR) has more recently expanded such potential, entailing further advantages related to enhanced dexterity, tremor filtration, and three-dimensional visualization, which facilitate precise parenchymal transection and vascular dissection [4–6].

Despite these advantages, intraoperative blood loss (IBL) remains one of the most relevant concerns in hepatic surgery since excessive bleeding may result in transfusion requirements, postoperative complications, and impaired oncological outcomes [7–9]. Intraoperative blood loss is influenced by patient characteristics, tumor location, and extent of resection [10, 11]. Nevertheless, few data are available on the determinants of blood loss in robotic liver resections; most of the evidence published so far comes from single-center or small retrospective series [12–14].

The present multicenter study aims to analyze IBL in robotic liver resections performed in nine European referral centers. The aims of the study were (1) to describe the perioperative outcomes of a large cohort of patients undergoing RALR, (2) to identify factors associated with increased intraoperative blood loss, and (3) to assess predictors of bleeding by means of multivariable analysis.

## Materials and methods

This retrospective multicenter cohort study included all patients who underwent robotic liver resection between January 2011 and December 2023 in nine high-volume European hepatobiliary centers. The data were anonymized, following the Declaration of Helsinki.

Demographic, clinical, intraoperative, and postoperative data were retrospectively collected from a shared database for each patient.

All consecutive patients who underwent (RALR) for benign or malignant liver lesions were enrolled in this study. The exclusion criteria for this study are as follows: (1) presence of portal hypertension; (2) hand-assisted procedures; (3) conversion to open surgery before parenchymal transection and (4) incomplete intraoperative data on blood loss.

Parenchymal transection was performed using standardized robotic techniques, including the MAMBA technique [15] or the Robolap approach [16]. Nevertheless, operative conduct and fine technical details remained dependent on the individual surgeon.

The following preoperative variables were collected: age, sex, body mass index (BMI), comorbidities, Charlson Comorbidity Index (CCI) score, ASA score, and tumor characteristics. Intraoperative variables collected included type and extent of resection (minor vs. major hepatectomy, anatomical vs. nonanatomical), operative time, use of Pringle maneuver, and IBL. Lastly, the postoperative outcomes included complications according to the Clavien-Dindo classification [17], length of stay, and 90-day mortality. Major complications were considered Clavien-Dindo ≥ 3, and in-hospital mortality was recorded according to the Clavien-Dindo classification (grade V).

Intraoperative blood loss was estimated by the anesthesiology team based on suction volumes minus irrigation fluids, in accordance with institutional practice. Although this approach reflects routine clinical assessment, it remains an approximation, as recommended by the recent HPB consensus on intraoperative blood loss reporting [18]. Patients were divided into two groups based on IBL according to the threshold value of 500 ml. This threshold was selected as it represents the commonly accepted level beyond which intraoperative blood transfusion is typically considered in hepatic surgery [18].

Continuous variables were expressed as mean ± standard deviation or median (interquartile range, IQR), as appropriate. Categorical variables were reported as frequencies and percentages. Comparisons between groups were made by t-test or Mann–Whitney U test for continuous variables and χ² or Fisher’s exact test for categorical variables.

Variables with a *p*-value < 0.10 in univariable analysis or considered clinically relevant were included in a multivariable logistic regression model to identify independent predictors of intraoperative blood loss ≥ 500 mL. Variables entered into the model included sex, cirrhosis, tumor size, previous open surgery, TAMPA score [19], and anatomical resection. Results were reported as odds ratios (ORs) with 95% confidence intervals (CIs). Statistical analyses were performed using SPSS software (IBM Corp., Armonk, NY, USA). A two-tailed p-value < 0.05 was considered statistically significant.

## Results

A total of 708 patients who underwent robotic liver resection were included across nine European centers. The mean patient age was 64.2 years, and 39.3% were female. The mean BMI was 26.6 kg/m². 48% of patients were classified as ASA ≥ III, and the mean CCI score was 6.15. Previous open abdominal surgery was reported in 48.2% and prior laparoscopic surgery in 23% of cases. Cirrhosis was present in 38.3% of the cohort, while 2.5% of patients were Child–Pugh class B. Preoperative chemotherapy was carried out in 27.1% of all cases. In 24.6%, there were multifocal lesions. The mean tumor diameter was 33.8 mm.

Totally, 49.4% of procedures consisted of anatomic resections, and the operative time was 251.9 min on average, with an IBL of 224.7 mL. Conversion to open surgery took place during 31.9% of all interventions, while intraoperative blood transfusion was applied in 4.5% of the patients. Pedicle clamping was utilized in 41.8%, and its duration was 41.8 min on average.

A postoperative complication occurred in 48.9% of patients, a major complication in 4.1%. In-hospital mortality occurred in 1% of patients. The mean hospital stay was 5.5 days, while the average length of ICU stay was 0.7 days.

All data are summarized in Table 1.

**Table 1:** Data on all patients

Variables	*n* = 708
Age (year), mean	64,2
Female, n (%)	278 (39,3)
BMI (kg/ m2), mean	26,59
ASA score ≥ III, n (%)	340 (48)
Charlson comorbidity score, mean	6,15
Previous open surgery, n (%)	341 (48,2)
Previous laparoscopic surgery, n (%)	163 (23)
Smoking, n (%)	134 (18,9)
Diabetes, n (%)	155 (21,9)
Cardiovascular disease, n (%)	248 (35)
Kidney failure, n (%)	28 (3,9)
Pulmonary disease, n (%)	58 (8,2)
Cirrhosis, n (%)	271 (38,3)
Child Score B, n (%)	18 (2,5)
Preoperative Chemotherapy, n (%)	192 (27,1)
Multifocal lesions, n (%)	174 (24,6)
Tumor size (mm), mean	33,8
TAMPA Score, mean	13,4
Lesion in contact with vessels, n (%)	217 (30,6)
Rehepatectomy, n (%)	53 (7,5)
Anatomical resection, n (%)	350 (49,4)
Lymphadenectomy, n (%)	69 (9,7)
Conversion to open, n (%)	226 (31,9)
Operative time (min), mean	251,9
Intraoperative blood loss (ml), mean	224,7
Blood transfusion, n (%)	32 (4,5)
Pedicule clamping, n (%)	296 (41,8)
Pedicle clamping duration (min), mean	41,8
Post-operative complication, n (%) Biliary leakage, n (%) Hemorrhage, n (%) Prolonged pain, n (%) Ascitis, n (%) Pulmonary infection, n (%) Other infections, n (%)	346 (48,9) 16 (2,3) 13 (1,8) 41 (5,8) 30 (4,2) 39 (5,5) 29 (4,1)
Clavien-Dindo ≥ 3, n (%)	29 (4,1)
Clavien-Dindo 5, n (%)	7 (1)
Reintervention, n (%)	5 (0,7)
ICU stay, mean	0,7
Total hospital stay, mean	5,5
90-day readmission, n (%)	34 (4,8)

BMI: body mass index; ASA: American society of anesthesiologists; ICU: intensive care unit

The patients were divided into two groups according to IBL: < 500 mL (*n* = 640, 90.4%) and ≥ 500 mL (*n* = 68, 9.6%), and data are summarized in Table 2.

**Table 2:** Comparison of the two groups

Variables	IBL < 500 ml *n* = 640	IBL 500 ≥ ml *n*= 68	*p*-value
Age (year), mean	64,2	64,36	0,903
Female, n (%)	261 (40,8)	17 (25)	0,013
BMI (kg/ m2), mean	26,5	27,1	0,247
ASA score ≥ III, n (%)	306 (47,8)	34 (50)	0,506
Charlson comorbidity score, mean	6,2	5,7	0,122
Previous open surgery, n (%)	301 (47)	40 (58,8)	0,074
Previous laparoscopic surgery, n (%)	147 (23)	16 (23,5)	0,881
Smoking, n (%)	126 (19,7)	8 (11,8)	0,142
Diabetes, n (%)	141 (22)	14 (20,6)	0,878
Cardiovascular disease, n (%)	230 (36)	18 (26,5)	0,141
Kidney failure, n (%)	23 (3,6)	5 (7,3)	0,177
Pulmonary disease, n (%)	50 (7,8)	8 (11,8)	0,247
Cirrhosis, n (%)	229 (35,8)	42 (61,8)	< 0,001
Child Score B, n (%)	15 (2,3)	3 (4,4)	0,404
Preoperative Chemotherapy, n (%)	179 (28)	13 (19,1)	0,151
Multifocal lesions, n (%)	158 (24,7)	16 (23,5)	0,821
Tumor size (mm), mean	33,1	40,2	0,013
TAMPA Class, mean	13,1	16,1	< 0,001
Lesion in contact with vessels, n (%)	198 (30,9)	19 (27,9)	0,142
Rehepatectomy, n (%)	50 (7,8)	3 (4,4)	0,466
Anatomical resection, n (%)	300 (46,9)	50 (73,5)	< 0,001
Lymphadenectomy, n (%)	65 (10,1)	4 (5,9)	0,387
Conversion to open, n (%)	218 (34,1)	8 (11,8)	0,004
Operative time (min), mean	239,1	372,5	< 0,001
Blood transfusion, n (%)	17 (2,6)	15 (22)	< 0,001
Pedicule clamping, n (%)	258 (40,3)	38 (55,9)	0,014
Pedicle clamping duration, mean	40,8	48,4	0,053
Post-operative complication, n (%) Biliary leakage, n (%) Hemorrhage, n (%) Prolonged pain, n (%) Ascitis, n (%) Pulmonary infection, n (%) Other infections, n (%)	297 (46,4) 14 (2,2) 7 (1,1) 37 (5,8) 24 (3,75) 27 (4,2) 25 (3,9)	49 (72) 2 (2,9) 6 (8,7) 4 (5,8) 6 (8,7) 12 (17,4) 4 (5,8)	< 0,001 0,660 < 0,001 1,000 0,058 < 0,001 0,513
Clavien-Dindo ≥ 3, n (%)	22 (3,4)	7 (10,3)	0,015
Clavien-Dindo 5, n (%)	4 (0,6)	3 (4,4)	0,022
Reintervention, n (%)	5 (0,8)	0	1,000
ICU stay, mean	0,7	1,1	< 0,001
Total hospital stay, mean	5,3	7,3	< 0,001
90-day readmission, n (%)	28 (4,4)	6 (8,8)	0,127

BMI: body mass index; ASA: American society of anesthesiologists; ICU: intensive care unit

There were fewer women in the IBL ≥ 500 mL group than in the IBL < 500 mL group (25% vs. 40.8%, *p* = 0.013), while more patients had cirrhosis (61.8% vs. 35.8%, *p* < 0.001). Mean tumor size was larger in the IBL ≥ 500 mL group (40.2 mm vs. 33.1 mm, *p* = 0.013) as well as mean TAMPA score (16.1 vs. 13.1, *p* < 0.001), reflecting greater procedural complexity.

The IBL ≥ 500 mL group underwent significantly more anatomical resections: 73.5% vs. 46.9%, *p* < 0.001. Mean operative time was longer in the IBL ≥ 500 mL group: 372.5 min vs. 239.1 min, *p* < 0.001. Conversion to open surgery was lower in this group: 11.8% vs. 34.1%, *p* = 0.004. A detailed breakdown of conversion indications was not uniformly available across centers and therefore could not be reliably analyzed.

Intraoperative transfusion was necessary in 22% of patients with IBL ≥ 500 mL compared with 2.6 of % IBL < 500 mL group, with a *p* < 0.001. Similarly, pedicle clamping was used more often, at 55.9% compared to 40.3%, with a *p* = 0.014.

The postoperative results were worse in the IBL ≥ 500 mL group, with a significantly higher overall complication rate (72% vs. 46.4%, *p* < 0.001) and more frequent major complications (10.3% vs. 3.4%, *p* = 0.015). In addition, postoperative hemorrhage (8.7% vs. 1.1%, *p* < 0.001), pulmonary infections (8.7% vs. 4.2%, *p* < 0.001), and other infectious complications (17.4% vs. 3.9%, *p* < 0.001) were also significantly increased. In-hospital mortality was higher among patients with IBL ≥ 500 mL (4.4% vs. 0.6%, *p* = 0.022).

The mean duration of ICU stay was 1.1 days in the IBL ≥ 500 mL group versus 0.7 days IBL < 500 mL group (*p* < 0.001). Total hospital stay was also higher in the IBL ≥ 500 mL group (7.3 days versus 5.3 days; *p* < 0.001).

Variables with *p* < 0.05 at the univariable analysis were entered into a multivariate logistic regression model to identify independent predictors of significant intraoperative blood loss (≥ 500 mL) (Table 3).

**Table 3:** Data after multivariate analysis

Variables	IBL < 500 ml *n* = 640	IBL ≥ 500 ml *n*= 68	*p*-value	
				95% CI
Female, n (%)	40,8	25	0,013	0.34–1.21
Cirrhosis, n (%)	35,8	61,8	< 0,001	1.63–5.52
Tumor size (mm), mean	33,1	40,2	0,013	0.99–1.02
Previous open surgery, n (%)	47	58,8	0,074	1.00-3.02
TAMPA Class, mean	13,1	16,1	< 0,001	1.03–1.16
Anatomical resection, n (%)	46,9	73,5	< 0,001	0.80–3.43

At multivariable logistic regression analysis (Table 3), cirrhosis was identified as an independent predictor of intraoperative blood loss ≥ 500 mL, increasing the risk approximately threefold (OR = 3.00; 95% CI 1.63–5.52; *p* < 0.001).

A higher TAMPA class was also independently associated with an increased risk of bleeding, with each one-point increase corresponding to a 9% higher probability of blood loss ≥ 500 mL (OR = 1.09; 95% CI 1.03–1.16; *p* = 0.004).

Previous open surgery showed a borderline association (OR = 1.74; 95% CI 1.00–3.02; *p* = 0.05).

Female sex, tumor size, and anatomical resection were not independently associated with increased intraoperative bleeding.

## Discussion

This large multicenter study analyzes intraoperative blood loss during robotic liver resection across nine European high-volume hepatobiliary centers, representing one of the largest collaborative experiences specifically focused on bleeding risk in robotic hepatectomy. Although robotic liver surgery has been associated with reduced blood loss compared with open and laparoscopic approaches in several series, intraoperative bleeding remains a relevant clinical issue, as excessive blood loss is known to negatively affect postoperative outcomes [1–9].

In the present cohort, the overall mean intraoperative blood loss was relatively low, yet nearly 10% of patients experienced blood loss ≥ 500 mL. This subgroup showed significantly worse perioperative outcomes, confirming that intraoperative bleeding remains clinically meaningful even in the robotic era. Among the factors analyzed, cirrhosis emerged as the strongest independent predictor of major blood loss. This finding is consistent with prior evidence from open and minimally invasive liver surgery and reflects the intrinsic vulnerability of cirrhotic parenchyma, altered vascular anatomy, and impaired hemostatic balance [10–14]. While robotic assistance may enhance precision, it does not fully offset the pathophysiological challenges associated with cirrhosis.

Procedural complexity, as measured by the TAMPA score, was the second independent determinant of significant blood loss. Increasing TAMPA scores were associated with a progressively higher bleeding risk, confirming that anatomical difficulty and proximity to major vascular structures remain key drivers of intraoperative hemorrhage [15–20]. Although the robotic platform may expand the technical feasibility of complex resections, it does not eliminate the intrinsic bleeding risk associated with demanding procedures, and TAMPA likely captures several anatomical and technical factors not otherwise measurable in large multicenter datasets.

Patients with blood loss ≥ 500 mL experienced longer operative times, higher morbidity, increased mortality, and prolonged ICU and hospital stays. These findings are in line with previous studies demonstrating a strong association between intraoperative bleeding and adverse postoperative outcomes in hepatic surgery [7–9]. The relationship between operative time and blood loss is likely bidirectional, as complex procedures predispose to both prolonged surgery and bleeding, while hemorrhagic events themselves may further extend operative duration [13].

Despite significant blood loss, only 22% of patients in this subgroup required intraoperative transfusion. This relatively low transfusion rate likely reflects the adoption of restrictive transfusion strategies in high-volume hepatobiliary centers, where transfusion decisions are guided by hemodynamic parameters and hemoglobin levels rather than estimated blood loss alone. Such an approach is supported by literature linking allogeneic transfusion to increased postoperative morbidity, particularly infectious complications, as well as potential adverse oncologic effects [21–24].

The overall conversion rate observed in this study was higher than that reported in some contemporary robotic liver surgery series. This finding should be interpreted in the context of the multicenter design and long inclusion period, which encompasses the early learning curve of several robotic programs [25–32]. Conversion thresholds are highly center- and surgeon-dependent and may reflect a proactive safety strategy rather than technical failure. The lower conversion rate observed in patients with higher blood loss is counterintuitive and likely reflects differences in conversion timing: early conversion in technically challenging cases may prevent major bleeding, whereas delayed conversion in complex resections may allow completion of the procedure robotically despite significant hemorrhage.

Several clinically relevant variables could not be incorporated into the multivariable analysis. None of the patients included had clinically significant portal hypertension, limiting its confounding role. Although standardized robotic techniques such as the MAMBA and Robolap approaches were adopted, operative conduct remained dependent on individual surgeon experience and intraoperative judgment. In addition, heterogeneity in anesthetic management—particularly regarding central venous pressure control and fluid administration—is expected in a multicenter cohort and represents an additional unmeasured source of variability that may have influenced blood loss [33–35].

The threshold of 500 mL used to define significant intraoperative blood loss deserves consideration. While any dichotomization of a continuous variable is inherently arbitrary, this cut-off was selected because it represents the level at which intraoperative transfusion is commonly considered in hepatic surgery, in accordance with recent consensus recommendations. Future studies may benefit from alternative analytic approaches, including sensitivity analyses or continuous modeling of blood loss.

Overall, these findings indicate that while robotic liver resection allows effective hemostatic control in most cases, significant bleeding still occurs in a meaningful minority of patients and is associated with worse postoperative outcomes. Identification of patient- and procedure-related risk factors should therefore inform preoperative planning and intraoperative decision-making, including case selection, vigilance during complex resections, and an appropriately low threshold for early conversion when required.

## Limitations

The limitations of this study, which must be taken into consideration, include the fact that, despite the prospective nature of the data sources, the retrospective nature of the analysis makes the study prone to selection and information bias. Although the sources have adopted common definitions, the accuracy of the reporting, especially regarding intraoperative variables, may vary.

Intraoperative blood loss was calculated based on the anesthesiology records, which estimate blood loss based on suction volumes and irrigation subtraction. This method is currently recommended by most experts. However, this method is an estimation, and the accuracy may vary depending on the intraoperative documentation habits at each center.

Although this study represents one of the largest series of robotic liver resections, the number of patients with significant blood loss was limited. Therefore, the number of patients with IBL ≥ 500 mL was limited, which restricted the complexity of the multivariate models. In addition, many factors that influence blood loss, such as central venous pressure management, pneumoperitoneum, anesthesia, and transection devices, were not uniformly documented.

Despite the standardization of the parenchymal transection techniques using the MAMBA and Robolap approaches, some degree of heterogeneity in the techniques used by the individual surgeons was inevitable.

Secondly, despite the advantages of the multicenter study design, the heterogeneity between centers was not formally accounted for in the analysis using the mixed-effects model. The low number of bleeding episodes and the unequal distribution of the data across the centers made the analysis less feasible, and the effect was controlled descriptively. Additionally, the detailed analysis of the causes of conversion was not possible due to the retrospective design of the study and the lack of standardization in the documentation of the causes of conversion across the centers.

## Conclusions

These results of this study confirm that, despite the technical benefits of robotic assistance, intraoperative blood loss is a clinically significant event with significant postoperative impact. Early recognition of patients at higher risk, especially those with cirrhosis, and complex procedures, should inform specific surgical approaches, such as careful patient selection, increased intraoperative caution, timely inflow control, and a low threshold for early conversion when indicated.

Robotic liver resection is a safe and effective procedure in experienced centers; however, the benefits of the procedure do not preclude the need for sound surgical judgment and context-driven decision-making. Future prospective multicenter studies are needed to better define risk factors, standardize intraoperative practices, and better understand the relationship between blood loss, timing of conversion, and long-term outcomes.

## Data Availability

The data presented in this study are available upon request from the corresponding author due to privacy reasons.
